# Relative Efficiency of Radiation Treatment Centers: An Application of Data Envelopment Analysis

**DOI:** 10.3390/healthcare10061033

**Published:** 2022-06-02

**Authors:** Tiffany Bayley, Mehmet A. Begen, Felipe F. Rodrigues, David Barrett

**Affiliations:** 1 Ivey Business School, University of Western Ontario, London, ON N6G 0N1, Canada; mbegen@ivey.uwo.ca (M.A.B.); dbarrett@ivey.ca (D.B.); 2 School of Management, Economics, and Mathematics, King’s University College, University of Western Ontario, London, ON N6A 2M3, Canada; frodrig7@uwo.ca

**Keywords:** cancer care, data envelopment analysis, relative efficiency, radiation treatment, continuous improvement

## Abstract

This study determines the relative efficiencies of a number of cancer treatment centers in Ontario, taking into account the differences among them so that their performances can be compared against the provincial targets. These differences can be in physical and financial resources, and patient demographics. An analytical framework is developed based on a three-step data envelopment analysis (DEA) model to build efficiency metrics for planning, delivery, and quality of treatment at each center. Regression analysis is used to explain the efficiency metrics and demonstrates how these findings can inform continuous improvement efforts.

## 1. Introduction

The role of radiotherapy in oncology has increased over the last decade as technological advances have continued [1,2]. Providing access to timely and appropriate radiotherapy services is crucial in order to minimize radiotoxicity and optimize patient outcomes [3,4,5].

In Ontario, Canada, there are 15 regional cancer centers (RCCs) that provide radiotherapy services to its 14.5 million residents. As a means of promoting access, RCCs are distributed across the Province and vary in size, availability of specialized equipment, and extent of clinical expertise [6]. While the provincial health authority devised a plan in 2015 to increase the performance of its cancer treatment centers through continuous improvement cycles, the challenge became how to compare, identify, and subsequently implement improvement opportunities and best practices to all its centers [7]. Given the heterogeneity of center attributes and available resources, measuring their relative performances against the same benchmarks may not be a fair assessment. For RCCs with a similar set of resources (inputs), one would expect similar levels of performance, including, for example, the number of patients completing treatment and the percentage of patients starting treatment within pre-specified wait-time targets (outputs). Similarly, one may expect an RCC with fewer inputs to yield lower performance. In reality, however, some RCCs may be more (or less) efficient, producing greater (or fewer) outputs with the same or fewer inputs due to a variety of reasons, such as patient composition, types of services provided, and whether the center is a teaching hospital or not.

Data envelopment analysis (DEA) is a linear programming technique that is widely used to compare decision-making units (DMUs) that provide similar services but operate with differing levels of resources. DEA enables an improved comparison of the relative efficiency of each DMU, that is, how well each DMU is able to transform its set of inputs into desired outputs. Following DEA, regression analysis can be used to identify factors associated with inefficiency, and how a less-efficient DMU can improve its performance [8,9,10,11,12]. (We use the terms DMU and RCC interchangeably).

This study employs DEA and regression analysis using data from 2013 to 2016 obtained from two provincial databases that report cancer-related patient-level activity and diagnoses. DEA is used to identify the factors associated with efficient planning and treatment of radiation therapy for patients treated for cancer in Ontario RCCs, and regression analysis is used to explain findings from the DEA model. We show how this analysis can be used in the development of continuous improvement initiatives, which is not typically discussed in DEA research. There is an opportunity to address gaps in the literature, specifically by contributing a DEA study focused on cancer care in a Canadian province with novel managerial insights regarding how to interpret and act upon efficiency scores. To the best of our knowledge, this is the first study to use DEA to evaluate radiation treatment center performance, and coupled with this are useful interpretations of model results that can initiate improvement efforts. With this study, more awareness is shone on challenges faced by provincial healthcare providers. We also demonstrate how integrated benchmarks can guide decision-makers and lead to potentially beneficial collaborations between RCCs.

## 2. Literature Review

Özcan [13], Canter and Poh [8], and Kohl et al. [14] explored DEA in healthcare settings, providing several examples of applications to hospitals, nursing homes, and international health studies. In particular, Cantor and Poh [8] reviewed articles that use DEA in combination with other technical approaches, such as regression or factor analysis, to measure healthcare system efficiency, and Kohl et al. [14] provided a review of DEA applied specifically in hospital settings. Those authors note an important disconnect between the findings of DEA and the action taken to improve efficiency: being able to identify low-performing DMUs and quantifying inputs (or outputs) that would yield 100% relative efficiency are important first steps; however, DEA does not prescribe a process for using that knowledge to achieve those targets.

Efficiency in hospitals has been studied before [10,15,16], along with the effectiveness of cancer screening programs as measured through detection rates [17,18] and cost [19,20]. However, few studies directly relate to our context, i.e., efficiency analysis of radiation centers. Langabeer and Özcan [21] applied DEA Malmquist in their longitudinal study of inpatient cancer centers across a five-year period in the United States, and uncovered that greater specialization of treatment does not necessarily lead to higher efficiency or lower costs. This was one of few DEA studies dedicated to cancer care. Meanwhile, Allin et al. [11] focused on comparing 89 health regions in Canada, examining the potential years of life lost that could be due to system inefficiencies, though their focus was not on cancer care.

Expanding on seminal work in DEA [22,23], Simar and Wilson [24] and Lothgren and Tambour [25] established bootstrapping frameworks for approximating a sampling distribution of the relative scale efficiencies, thereby enabling the construction of confidence intervals for these estimates. Bootstrap DEA can be applied to any industry sector, from banking [26] to education [27], to and healthcare supply chains [28]. In each of these settings, regression analysis follows DEA to identify potential causes of inefficiency for the DMUs.

## 3. Problem Description

Each of the 15 cancer centers in Ontario is subject to specific levels of available resources and expertise (Table 1). For example, some programs are located in teaching hospitals and others are not, and each center has its own level of specialized equipment required for radiotherapy, or medical resource level. Centers can be further distinguished by their treatment capabilities, or their “diversification”. It was observed that for every center, the majority of treatments delivered were to the pelvis and chest, but certain centers had wider or more *diversified* “portfolios” of body regions treated (e.g., brain). This is in contrast to *specialization*, which implies that a center would treat only certain body regions and not others. We express diversification as the proportion of radiation treatments delivered to body regions other than pelvis or chest. Finally, centers are situated across the province, where local populations vary. Catchment population refers to the census population within a 50-kilometer radius of the RCC as determined by population counts reported in the 2011 and 2016 census reports [29,30]. Annual population growth rates for Canada [31] were used to estimate populations between 2012 and 2015, inclusive.

Measuring the performances of these distinct centers against the same benchmarks may not be the best assessment. Rather, their performances *relative to their available inputs and respective outputs* would provide a clearer picture of how efficiently they are operating compared to one another. The scope of our study is limited to cancer treatments delivered by linear accelerators, or LINACs.

**Figure 1:**
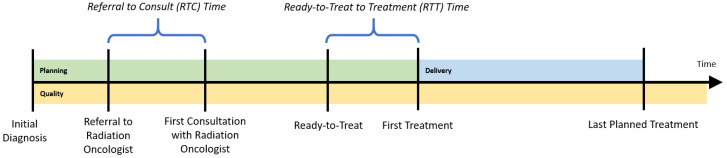
Key dates.

To determine the appropriate inputs and outputs for the regional cancer centers (RCCs), a patient’s radiation treatment journey is followed, while considering three dimensions of treatment: *planning*, *delivery*, and *quality*. The planning dimension begins with the patient’s initial diagnosis date. The patient is then referred for a consultation with a radiation oncologist. The time between referral and the first consultation visit with a radiation oncologist is an important performance indicator, called referral-to-consult (RTC). If radiotherapy is indicated, the radiation oncologist will then develop a course of treatment, and will also determine the date that treatment could begin (the ready-to-treat date). The time between when the patient is physically ready to be treated and their first treatment is another important indicator, the ready-to-treat to first treatment (RTT) time. To monitor wait times for the radiation treatment program, the provincial health authority established a 14-day benchmark for both the RTC and RTT wait times for all RCCs; the proportion of patients whose wait time is within that target is tracked yearly. While each patient’s course of treatment (e.g., dosages and timing of treatments) could differ, this planning phase should be consistent for all patients (Figure 1).

Once a plan is in place, radiation treatments are administered according to that plan (the *delivery* dimension of treatment). This requires patients to visit their respective regional cancer centers on specific dates and times (e.g., radiation is applied Monday through Friday for 3 weeks). The number and timing of treatments that can be delivered at a center depend upon that center’s availability and utilization of its resources, which comprise medical equipment such as LINACs.

Patient support visits and quality assurance visits could also be booked around these treatment visits to minimize the number of center visits required of the patient. Patient support visits consist of patient education and coordination/scheduling of radiation-related visits. Quality assurance visits include exposing the patient to a thermoluminescent dosimeter, acquisition of portal images or volumetric images, use of active breathing control, use of respiratory gating equipment, manual calculations, fluence/dosimetry checks and peer review. These activities are captured in the *quality* dimension of treatment planning and delivery. Figure 1 illustrates the approximate timing of each treatment dimension. Though several visits may occur simultaneously without requiring the patient to physically change locations within the center, we consider them to be distinct. This allows for resources, such as number of staff, technicians, and physicians, to be indirectly incorporated in the DEA model, should information pertaining to full-time equivalent levels not be easily accessible.

### 3.1. Data

Data between 2013 and 2016 were examined from two databases: Activity Level Reporting (ALR), which reports patient-level activity within the cancer system, and Ontario Cancer Registry (OCR), a database of residents in Ontario who have been diagnosed with cancer and residents who have died of cancer. From ALR, total number of visits by type (e.g., planning and simulation) and number of incident cases (i.e., number of new cases of diagnosed cancer) were gathered. The patient was assigned to an RCC based on the location of their first treatment. Number of deaths by year and by RCC was calculated by linking to the OCR and the Registered Persons Database, which hold information on Ontario residents’ access to public health services. The proportion of patients whose RTC and RTT wait times were within the provincial target of 14 days was calculated, along with total number of radiation treatments delivered to specific body regions. From both ALR and OCR, number of new cancer diagnoses after the first initial diagnosis for a patient as a surrogate for the subsequent cancer diagnosis rate was identified. Publicly available sources for teaching hospital designations were consulted [32], and they were for data on medical resource (MR) capacity and utilization too [33,34].

### 3.2. Privacy and Software

This study was approved by the Western University Health Sciences Research Ethics Board. Data envelopment analysis was performed using optimization solver CPLEX 12.10 via the Python API (Python version 3.6) and regression analysis was performed with statistical software R (version 3.6.0).

## 4. Computing Relative Efficiencies

To compare the performances of RCCs between 2013 and 2016, a DEA model to compute the relative efficiency score for each center across the three treatment dimensions each year was constructed. We posit that the provincial health authority has more control over its inputs than outputs and that resources are limited in our problem setting (as is typical in healthcare environments), so the DEA was formulated as an input-oriented, variable-returns-to-scale (VRS) model, where economies of scale may exist (i.e., we do not assume there is a constant rate of substitution between inputs and outputs). In other words, the VRS assumption is more general and does not require that a change in the inputs produce a proportional change in the outputs. Details regarding the mathematical formulation of the input-oriented VRS DEA model, [DEA], can be found in Appendix A.1, and we note once again that RCCs are analogous to decision-making units (DMUs).

**Figure 2:**
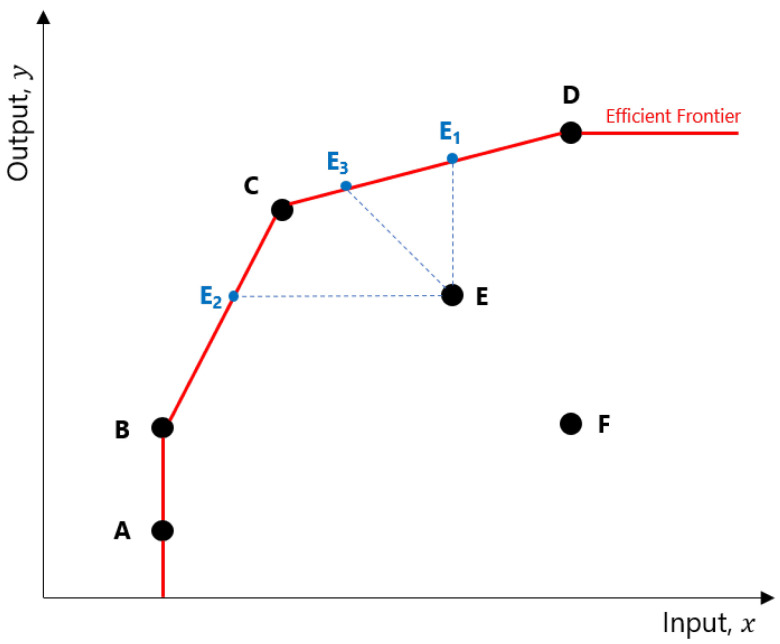
Visualization of a VRS model with a single input and single output.

The relative efficiencies computed in a DEA model indicate how well an RCC is able to transform its inputs into outputs, relative to other RCCs. Consider Figure 2. The points A through F represent different RCCs and the levels of output they can achieve based on their inputs. The DEA determined that RCCs A, B, C, and D are efficient, and joining their coordinates will form the *efficient frontier*. An inefficient RCC such as E can improve its relative efficiency in several ways: increase output without changing its original input (move to E_1_), use fewer inputs to produce the same level of outputs (E_2_), or adjust both inputs and outputs in such a way as to reach the frontier (E_3_).

Efficiency scores for the planning dimension were generated using the numbers of clinic visits, planning visits, and simulation visits as inputs; and the percentages of patients with RTC and RTT times ≤14 days as outputs (Table 2). Efficiency scores for the delivery dimension used medical resource (MR) capacity and the inverse of RTT times ≤14 days as inputs; and MR utilization and the number of treatment visits as outputs. The *inverse* of RTT was used because a smaller input value is desirable in an input-oriented DEA model (i.e., a lower inverse RTT implies that a higher proportion of patients are within the target wait time window). Similarly, a larger output value is desirable in this type of analysis.

Lastly, efficiency in the quality dimension used the number of patient support visits, quality assurance visits, and treatment visits as inputs, and survival rate and the inverse of the subsequent diagnosis rates as the outputs. The subsequent diagnosis rate was measured by first identifying for each patient in the population over all years in our study whether an incident (i.e., cancer diagnosis) was the first or a subsequent cancer diagnosis. Then, in a given year, the total number of subsequent cancer diagnoses across the patient population was divided by the total number of cancer incidents to determine the subsequent diagnosis rate. The complement of this value is used in this analysis, since larger outputs are more desirable in an input-oriented VRS model.

Solving [DEA] provides only a snapshot of relative efficiency scores, based on single measures of inputs and outputs at a given point in time. To better capture the variability in these efficiencies, a bootstrap DEA [24,35] allows computation of an average efficiency and confidence interval through sampling with replacement. This bootstrap approach (presented in Appendix A.2) repeatedly samples inputs to generate a range of efficiencies, ultimately providing an *average* efficiency score that better distinguishes efficient RCCs from one another, even if the differences are small. Table 3, Table 4 and Table 5 show efficiency scores derived from [DEA], along with the bias-corrected mean efficiencies and their 5th and 95th quantiles (denoted by θ, $\hat{\theta }$, $q_{0.05}$, and $q_{0.95}$, respectively). The ranking of RCCs remains, in general, consistent with results from solving [DEA] just once, so we see that results are robust.

## 5. Explaining Relative Efficiencies

We want to understand the differences in efficiencies to gain insights on how to interpret them. External factors that may be determinants of relative efficiency include:

(1) Center size;
(2) Center catchment population;
(3) Radiation treatment diversification;
(4) Teaching hospital designation.

Center size was estimated using the medical resource level (MRL) as a proxy (Table 1). Catchment population and radiation treatment diversification were defined in Section 3. Table 6 presents the variables in our regression model, and shows mean and standard deviation values for these regression variables over the four years of study.

The regression model takes the following form:

(1) $${\hat{\theta }}_{j}^{*}=\beta_{0}+\beta_{1}{\mathrm{MRL}}_{j}+\beta_{2}{\mathrm{DIV}}_{j}+\beta_{3}{\mathrm{POP}}_{j}+\beta_{4}{\mathrm{TEA}}_{j}+\epsilon_{j}$$

where ${\hat{\theta }}_{j}^{*}$ denotes a transformed bias-corrected efficiency score. As ${\hat{\theta }}_{j}$ are censored at 1, (i.e., $0\leq {\hat{\theta }}_{j}\leq 1$), this requires various cut-off points (and thus regression models) to be developed to compute meaningful efficiency estimates that remain within 0 and 1. However, by transforming scores so that they are left-censored at 0 only, a censored (or Tobit) regression can be applied and interpreted similarly to an ordinary least squares regression [13]. We apply the following transformation to compute ${\hat{\theta }}_{j}^{*}$:

(2) $${\hat{\theta }}_{j}^{*}=\frac{1}{{\hat{\theta }}_{j}}-1$$

Note that with this transformation, ${\hat{\theta }}_{j}^{*}\geq 0$ and can be interpreted as an “inefficiency” score; i.e., ${\hat{\theta }}_{j}^{*}=0$ indicates that RCC *j* is 100% efficient.

For each model dimension, a left-censored (Tobit) regression analysis was performed with the *vglm* function from the *VGAM* library in R using the transformed efficiencies over all four years of our study. From the results in Table 6, this combination of determinants explains roughly 64% and 38% of variation in the planning and quality efficiency scores, respectively, but does not adequately account for variation in delivery efficiency ($R^{2}<0.1$%).

Due to the transformation applied to efficiency scores, interpretation of coefficients must be handled carefully: a positive coefficient indicates that efficiency *worsens* as the dependent variable increases, and vice versa. For the *planning* dimension, higher efficiency scores were associated with smaller centers (lower levels of medical resources) (p<0.01) and centers with less diverse radiation treatment portfolios (p<0.001), but not catchment populations (p=0.253) or teaching designation (p=0.218). Smaller RCCs were also more efficient in the *quality* (p<0.05).

## 6. From Rankings to Continuous Improvement

Treatment center policies can be informed by relative efficiency scores. Rather than focusing solely on the rankings that a DEA provides, we want to work towards actionable plans that contribute to the continuous improvement of RCCs. We developed several visualizations to identify potential courses of action for an RCC to improve upon its performance in a given dimension. In the following subsections, visualizations for a selection of computed results are presented; similar analysis can be performed for all dimensions, years, and combinations of RCCs.

### 6.1. Comparing Results by Dimension

**Figure 3:**
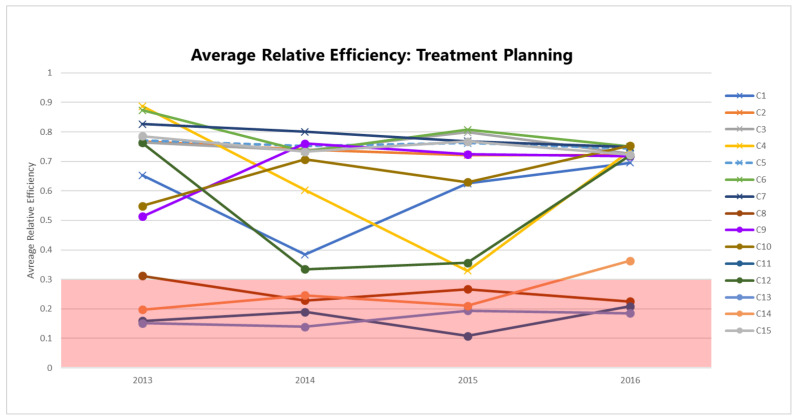
Mean bias-corrected planning efficiency scores.

For each of the three treatment dimensions, relative efficiency scores can be compared to identify whether any trends in performance are apparent. Figure 3 plots the bias-corrected mean efficiencies for the planning dimension ($\hat{\theta }$), computed according to the method in Appendix A.2 and shown in Table 3 by year and RCC.

**Figure 4:**
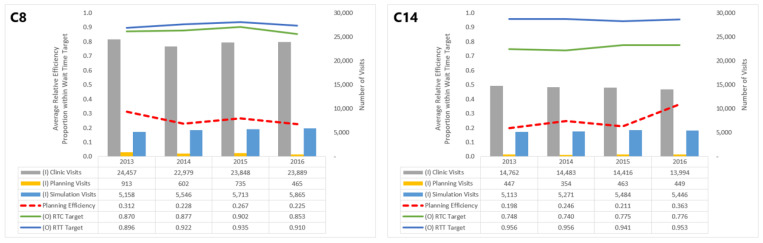
Comparing planning dimension performance: C8 and C14.

The efficiencies of some centers are distinctly lower than others: C8, C11, C13, and C14 consistently perform with $\hat{\theta }<0.30$. Focusing on a specific center or group of centers within this range, say C8 and C14, allows us to understand which inputs and outputs impact their planning efficiency scores (Figure 4).

Though both in the band of low efficiency, C14’s performance in the planning dimension has improved since 2013, whereas C8’s has seen a decline. These trends can be attributed numerous changes in inputs and outputs for C14 and C8. In particular, C14 saw a general decline in clinic visits and improvement in the proportion of patients meeting the RTC wait time target, which both outweigh the increase in simulation visits. For C8, however, while the number of planning visits decreased over this period, it did not offset increases in clinic and simulation visits or the worsening of the RTC target.

We do not recommend exhausting pairwise comparisons of centers; rather, decision-makers should identify “peer centers” (or, RCCs with similar performances in a treatment dimension) and scrutinize why those RCCs perform differently. In what specific measures does one RCC outperform the other, and how can each RCC strive for improvement?

### 6.2. Relative Comparison of RCCs

**Figure 5:**
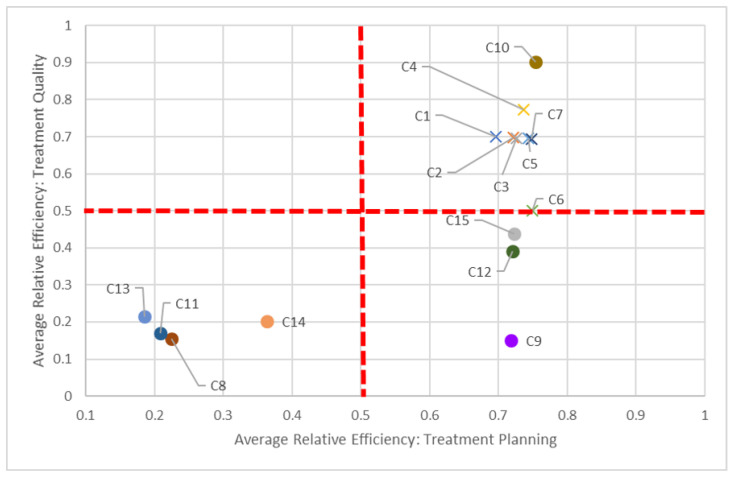
Benchmarking according to planning and quality dimensions (2016).

**Figure 6:**
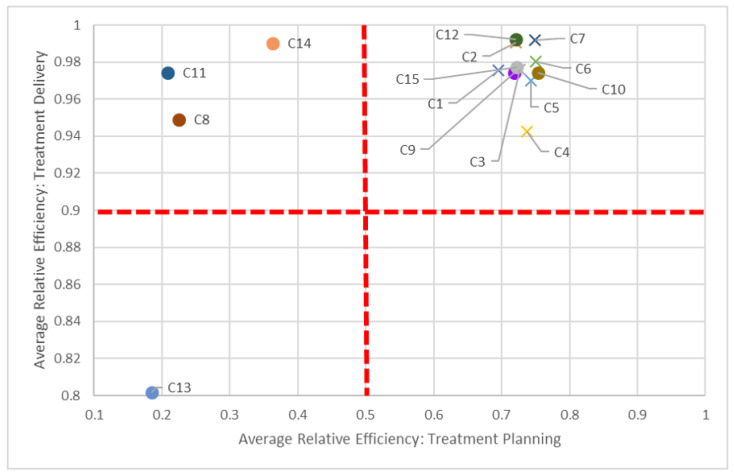
Benchmarking according to planning and delivery dimensions (2016).

**Figure 7:**
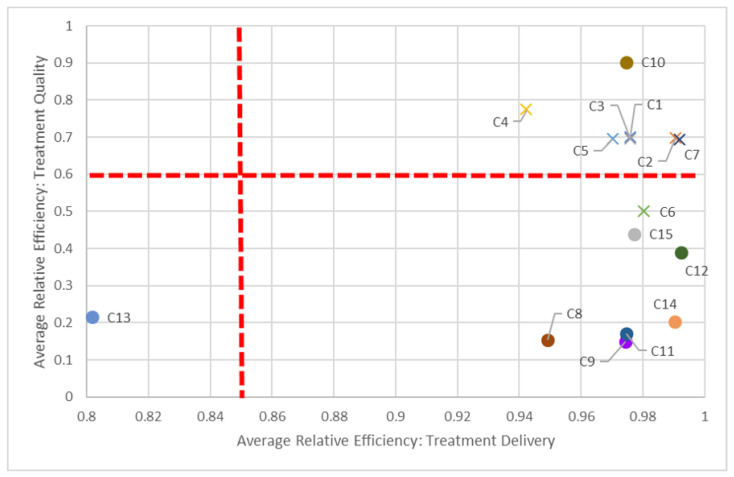
Benchmarking according to delivery and quality dimensions (2016).

To assist in identifying these peer centers, we propose visualizations in Figure 5, Figure 6 and Figure 7 to compare two dimensions in a given year (2016).

Say we compare centers based on both planning and quality dimensions (Figure 5). In keeping with our C8 and C14 pairing, we can see how “far” away C8 is compared to C14, but also how close it is in performance to other centers, such as C11. While all centers should be striving to be in the top-right quadrant (i.e., 100% efficient in both dimensions), in the short-term, it is perhaps better to compare just certain groups of centers, quadrant-by-quadrant. This visualization also quickly identifies clusters of centers: According to this study, non-teaching hospitals (denoted by “×”) performed better in both planning and quality in 2016, in general, compared to their teaching counterparts. This clustering is also apparent when comparing planning and delivery dimensions (Figure 6) and delivery and quality dimensions (Figure 7) in 2016, as most non-teaching hospitals performed better than non-teaching hospitals in all three dimensions.

The dotted red lines in each of these quadrant charts represent arbitrarily chosen thresholds for performance, and should be adjusted appropriately by decision-makers. For example, in Figure 7, if a 60% efficiency threshold in the quality dimension is considered too high to strive for, it can be lowered to some other value, say 30%. By focusing on fewer centers who have not met this 30% target, decision-makers can tailor strategic plans for those centers to get them past this milestone. Alternatively, centers scoring very high on quality (given managerial targets) may reduce efforts in this dimension to focus on under-performing aspects in other dimensions. Through these positive incremental changes, centers maybe be encouraged to adopt more continuous improvement initiatives.

## 7. Discussion

As part of measuring the performances of RCCs, the provincial health authority measures and reports various indicators, including RTC and RTT. Although this is an important and effective means of assessing improvement opportunities, indicators measured in isolation do not speak to the center’s efficiency. DEA, in contrast, provides a single numeric value that signifies each center’s relative efficiency and can identify system-level strategies towards performance improvement (e.g., increase medical resource levels).

We recognize that computing these scores is not enough; we must also consider how to influence managerial action from those scholarly insights [14]. Typically, inefficient RCCs would seek to reduce their inputs while maintaining or increasing their outputs, with specific targets obtained using slack values from model [P]. These RCCs may also consider emulating a “weighted” combination of efficient RCCs based on results from model [DEA]. While knowing these target values is useful, how to achieve them operationally is another story. We present a variation of this benchmarking information to assist in identifying potential partnerships between centers. Visualizing DEA inputs, outputs, and resulting efficiency scores allows policy makers to quickly see how centers are performing across several radiation treatment dimensions and against other centers over time. By considering three separate treatment dimensions, we show that an RCC that is inefficient in one dimension can also be a leader in another. The parameters listed in Table 2 were used as inputs and outputs. While there are other measures that could be incorporated, the number of RCCs we studied was a limiting factor: Özcan [36] suggests that the number of inputs and outputs to consider in a DEA model should satisfy n≥max(r×s,3(r+s)), where n,r, and *s* denote the numbers of RCCs, inputs, and outputs, respectively. With n=15 RCCs, we used r≤3 and s≤2 for any one DEA model.

Some determinants of relative efficiencies discussed in Section 5 are not within the decision-making jurisdiction of an individual radiation treatment center. For example, center catchment population and teaching hospital designation cannot be controlled or changed by an RCC. Medical resource levels and treatment diversification, however, can be influenced by the specific needs of the population served by the RCC and the policies developed by radiation treatment center administrators.

Furthermore, the inputs and outputs used in our analysis were limited to values that are currently available. Other center-specific information that could have been more informative would include full-time equivalents (FTEs) for radiation treatment professionals, such as oncologists, dosimetrists, technicians, and administrators. Provincial funding also differs by center and is partly based on the center’s case mix, neither of which was provided by the health authority for use in our study. A center’s case mix would influence the complexity of radiation treatments planned and delivered, which could impact RTC and RTT wait times, medical resource utilization, and patient survival rates, but also the specialization of FTEs and types of equipment required to deliver specialized treatments. Without this more granular information, insights from DEA models are limited to high-level interpretations.

Finally, it is important to be mindful of metrics deemed important by provincial authorities to ensure the insights developed by DEA modeling are meaningful. For example, during the analysis period (2013 to 2016), RTC and RTT wait times were considered important indicators for how well RCCs were meeting provincial wait time targets. However, in their most recent plan, the provincial authority modified these indicators to measure instead wait time from diagnosis to first treatment date and the radiation integrated wait time [37]. The DEA model should be revised to reflect these updates in provincial measurements. Regardless, the analytical approach we presented here can be followed with the appropriate value substitutions [10,11,21].

## 8. Conclusions

Using data envelopment analysis, multiple and varying inputs and outputs were considered together by separating the patient’s radiation treatment journey into three phases: planning, delivery, and quality. With bias-corrected DEA scores computed from the bootstrap model, efficient centers are better distinguished from one another based on their mean efficiencies compared to using VRS DEA results directly. The censored (Tobit) regression analysis identifies external determinants of efficiency, namely, center size (measured by medical resource level), diversification of radiation treatment, center catchment population, and teaching hospital designation. These determinants account for roughly 64% and 38% of variation in efficiencies in the planning and quality dimensions, respectively (but they did not significantly explain variation in the delivery dimension).

We highlight that this analysis is not prescriptive: while it can identify problem areas, it does not actually prescribe the specific actions for centers to take to reach their targets. Rather, with a larger number of RCCs for comparison, it can point decision-makers in directions that could lead to learning opportunities and beneficial collaborations between regional cancer centers for meeting provincial goals.

There are interesting empirical and theoretical future research directions for our work. For example, how can we combine three different efficiency scores into a single measure and evaluate all the centers with this new score? A single score will be more practical and easier to understand and act upon. However, building such a theoretical and empirical framework is an open research question. Another option would be to compare all centers within a province to those in another province while taking into account provincial differences. This would allow policy makers to improve centers even more.

## Figures and Tables

**Table 1:** Characteristics of regional cancer centers in Ontario.

RCC	Teaching Hospital (Yes =1)	Medical Resource Level ^a^	Diversification % ^a^	Catchment Population (’000s) ^a^
C1	0	[1, 6) ^b^	32.62	≤500 ^b^
C2	0	[6, 10)	21.28	>500
C3	0	[6, 10)	23.52	>500
C4	0	[6, 10)	20.06	>500
C5	0	[1, 6)	23.33	≤500
C6	0	[1, 6)	16.33	>500
C7	0	[6, 10)	21.01	≤500
C8	1	≥10	33.63	≤500
C9	1	≥10	36.49	>500
C10	1	[1, 6)	31.50	≤500
C11	1	≥10	55.69	>500
C12	1	[6, 10)	31.80	≤500
C13	1	≥10	40.13	≤500
C14	1	≥10	39.70	≤500
C15	1	[6, 10)	30.42	≤500

^a^ Yearly average from 2013 to 2016. ^b^ Values presented in this table have been categorized to maintain center anonymity; each center’s precise values were used in our quantitative analysis.

**Table 2:** Input and output parameters for DEA models.

Parameter	Description	Dim (I or O) ^1^		2013		2014		2015		2016	
				Mean	SD	Mean	SD	Mean	SD	Mean	SD
Clinic Visits	Number of new radiation and follow-up clinic visits with a physician	P(I)		12,568.87	7615.68	13,256.07	7458.43	13,910.4	7832.72	13,357.07	7782.20
Planning Visits	Number of radiation planning visits	P(I)		961.87	1307.27	889.40	1229.06	929.20	1313.66	939.53	1404.12
Simulation Visits	Number of visits involving conventional simulation, CT simulation, or emerging imaging methods	P(I)		3950.87	2450.01	3989.93	2730.23	3999.93	2505.11	4103.20	2479.76
RTC Target	% of patients whose time from referral to a radiation oncologist until the consult occurred (referral-to-consult; RTC) was ≤14 days	P(O)		82.81	8.29	86.51	6.39	87.20	6.58	85.67	6.14
RTT Target	% of patients who started treatment ≤14 days from the date the patient was deemed ‘ready-to-treat’ (RTT) by the radiation oncologist responsible for that patient’s care	P(O)	D(I) ^2^	93.58	4.42	94.03	3.21	92.81	3.44	90.74	7.17
MR Capacity	Available MR equipment hours	D(I)		21,310.40	11,790.95	21,310.40	11,790.95	21,310.40	11,790.95	19,136.80	12,658.32
MR Utilization	% time MR equipment is in use, calculated as the number of hours MR equipment was in use divided by MR capacity	D(O)		89.60	15.08	90.60	15.38	91.60	15.65	81.53	13.38
Treatment Visits	Number of visits where radiation is given with a LINAC	D(O)	Q(I)	48,462.67	38,107.06	48,491.93	39,037.90	50,253.33	38,813.57	49,702.87	37,659.65
Patient Support Visits	Patient education and co-ordination/scheduling of radiation-related visits	Q(I)		39,085.73	34,866.61	41,535.40	38,200.28	44,525.00	39,306.04	43,830.27	40,144.35
Quality Assurance Visits	Some activities include image acquisition, use of respiratory gating equipment, peer review, and fluence/dosimetry checks	Q(I)		77,278.80	91,423.45	79,555.27	87,393.01	84,650.60	89,687.21	87,858.20	91,477.60
Survival Rate (%)	1 − (Deaths ÷ Incidents)	Q(O)		81.91	17.80	81.07	18.14	84.95	14.90	81.31	17.74
Inverse Subsequent Diagnosis Rate (%)	1 − (Subsequent Cancer Diagnoses ÷ Incidents)	Q(O)		85.42	1.64	84.08	1.64	78.35	2.17	78.61	2.24

^1^ Dim = dimension, I = input, O = output; ^2^ inverse of rtt target was used.

**Table 3:** Planning dimension efficiencies, bias-corrected bootstrap efficiencies, and quantiles.

DMU	2013				2014				2015				2016			
	θ	$\hat{\theta }$	$q_{0.05}$	$q_{0.95}$	θ	$\hat{\theta }$	$q_{0.05}$	$q_{0.95}$	θ	$\hat{\theta }$	$q_{0.05}$	$q_{0.95}$	θ	$\hat{\theta }$	$q_{0.05}$	$q_{0.95}$
C1	0.743	0.653	0.538	0.736	0.454	0.384	0.289	0.451	0.738	0.625	0.506	0.722	0.838	0.696	0.605	0.808
C2	1.000	0.768	0.557	0.990	1.000	0.740	0.544	0.983	1.000	0.721	0.535	0.976	1.000	0.721	0.568	0.957
C3	1.000	0.765	0.546	0.991	1.000	0.737	0.544	0.983	1.000	0.798	0.587	0.972	1.000	0.727	0.570	0.959
C4	1.000	0.887	0.754	0.992	0.688	0.602	0.507	0.677	0.388	0.329	0.274	0.382	1.000	0.737	0.590	0.952
C5	1.000	0.771	0.567	0.991	1.000	0.752	0.580	0.985	1.000	0.762	0.629	0.976	1.000	0.744	0.616	0.953
C6	1.000	0.874	0.709	0.993	1.000	0.733	0.544	0.983	1.000	0.807	0.673	0.978	1.000	0.750	0.627	0.955
C7	1.000	0.826	0.637	0.992	1.000	0.800	0.614	0.984	0.938	0.769	0.603	0.916	1.000	0.749	0.615	0.953
C8	0.228	0.198	0.152	0.226	0.285	0.246	0.197	0.281	0.250	0.211	0.168	0.245	0.445	0.363	0.310	0.431
C9	0.342	0.312	0.263	0.340	0.264	0.228	0.193	0.260	0.307	0.267	0.232	0.300	0.280	0.225	0.194	0.267
C10	0.585	0.513	0.370	0.580	1.000	0.761	0.563	0.981	1.000	0.724	0.535	0.973	1.000	0.718	0.568	0.948
C11	0.602	0.548	0.453	0.597	0.792	0.706	0.619	0.779	0.731	0.629	0.545	0.713	1.000	0.754	0.632	0.955
C12	0.176	0.159	0.133	0.175	0.213	0.189	0.168	0.210	0.128	0.108	0.088	0.126	0.255	0.208	0.181	0.244
C13	1.000	0.763	0.546	0.991	0.384	0.334	0.267	0.381	0.422	0.357	0.285	0.416	1.000	0.720	0.569	0.952
C14	0.168	0.152	0.121	0.167	0.158	0.140	0.114	0.156	0.213	0.194	0.171	0.211	0.230	0.185	0.155	0.224
C15	0.885	0.786	0.633	0.878	1.000	0.733	0.544	0.983	0.891	0.768	0.582	0.874	1.000	0.722	0.568	0.948

**Table 4:** Delivery dimension efficiencies, bias-corrected bootstrap efficiencies, and quantiles.

DMU	2013				2014				2015				2016			
	θ	$\hat{\theta }$	$q_{0.05}$	$q_{0.95}$	θ	$\hat{\theta }$	$q_{0.05}$	$q_{0.95}$	θ	$\hat{\theta }$	$q_{0.05}$	$q_{0.95}$	θ	$\hat{\theta }$	$q_{0.05}$	$q_{0.95}$
C1	1.000	0.968	0.867	0.999	1.000	0.970	0.913	0.998	1.000	0.970	0.903	0.998	1.000	0.976	0.865	1.000
C2	0.988	0.978	0.966	0.986	0.981	0.970	0.954	0.980	1.000	0.987	0.973	0.999	1.000	0.990	0.975	1.000
C3	1.000	0.978	0.937	0.999	1.000	0.975	0.935	0.998	1.000	0.974	0.931	0.999	1.000	0.976	0.836	1.000
C4	0.907	0.899	0.889	0.906	0.924	0.919	0.912	0.923	0.942	0.936	0.929	0.941	0.945	0.942	0.938	0.945
C5	0.985	0.977	0.965	0.984	0.981	0.967	0.948	0.979	0.986	0.978	0.963	0.985	0.976	0.970	0.952	0.976
C6	0.980	0.969	0.947	0.979	1.000	0.981	0.960	0.999	0.886	0.875	0.848	0.885	1.000	0.980	0.912	1.000
C7	0.963	0.955	0.946	0.962	0.975	0.966	0.955	0.974	0.974	0.965	0.955	0.973	0.996	0.992	0.979	0.996
C8	0.926	0.919	0.912	0.925	0.953	0.945	0.936	0.952	0.977	0.970	0.961	0.976	0.951	0.949	0.944	0.951
C9	0.997	0.987	0.964	0.996	0.997	0.985	0.964	0.995	1.000	0.989	0.971	0.999	0.980	0.974	0.959	0.980
C10	1.000	0.987	0.960	0.999	1.000	0.985	0.947	0.999	1.000	0.986	0.953	0.999	1.000	0.974	0.836	1.000
C11	1.000	0.967	0.867	0.999	1.000	0.969	0.913	0.999	1.000	0.970	0.904	0.998	1.000	0.974	0.837	1.000
C12	1.000	0.986	0.973	0.999	0.911	0.902	0.890	0.910	0.943	0.934	0.923	0.941	1.000	0.992	0.978	1.000
C13	0.840	0.835	0.829	0.839	0.898	0.891	0.883	0.897	0.904	0.897	0.890	0.903	0.804	0.802	0.799	0.803
C14	0.984	0.975	0.963	0.983	0.982	0.972	0.958	0.981	0.978	0.969	0.956	0.977	0.992	0.990	0.987	0.992
C15	0.969	0.962	0.953	0.968	0.967	0.961	0.954	0.965	0.982	0.976	0.968	0.981	0.980	0.977	0.971	0.980

**Table 5:** Quality dimension efficiencies, bias-corrected bootstrap efficiencies, and quantiles.

DMU	2013				2014				2015				2016			
	θ	$\hat{\theta }$	$q_{0.05}$	$q_{0.95}$	θ	$\hat{\theta }$	$q_{0.05}$	$q_{0.95}$	θ	$\hat{\theta }$	$q_{0.05}$	$q_{0.95}$	θ	$\hat{\theta }$	$q_{0.05}$	$q_{0.95}$
C1	1.000	0.719	0.567	0.958	1.000	0.713	0.551	0.986	1.000	0.673	0.556	0.924	1.000	0.700	0.554	0.966
C2	1.000	0.680	0.531	0.958	1.000	0.711	0.550	0.984	1.000	0.698	0.577	0.933	1.000	0.698	0.548	0.965
C3	0.656	0.502	0.369	0.633	1.000	0.711	0.551	0.981	1.000	0.667	0.554	0.921	1.000	0.696	0.546	0.964
C4	1.000	0.676	0.514	0.962	1.000	0.776	0.561	0.985	1.000	0.673	0.558	0.926	1.000	0.773	0.571	0.967
C5	1.000	0.679	0.519	0.957	1.000	0.714	0.551	0.983	1.000	0.672	0.556	0.921	1.000	0.696	0.547	0.960
C6	0.249	0.193	0.142	0.243	0.817	0.693	0.516	0.805	0.745	0.572	0.482	0.694	0.641	0.500	0.399	0.621
C7	1.000	0.696	0.544	0.964	1.000	0.810	0.573	0.984	1.000	0.674	0.561	0.929	1.000	0.695	0.548	0.960
C8	0.767	0.583	0.433	0.747	0.192	0.165	0.137	0.189	0.228	0.168	0.139	0.211	0.192	0.154	0.117	0.188
C9	0.055	0.043	0.033	0.054	0.224	0.197	0.169	0.220	0.214	0.165	0.142	0.200	0.165	0.150	0.132	0.164
C10	0.407	0.357	0.301	0.403	0.910	0.841	0.760	0.905	1.000	0.794	0.685	0.927	0.998	0.903	0.802	0.988
C11	0.243	0.188	0.144	0.233	0.232	0.194	0.145	0.228	0.225	0.167	0.135	0.216	0.215	0.170	0.125	0.211
C12	0.306	0.229	0.175	0.294	0.372	0.341	0.305	0.366	0.411	0.323	0.277	0.394	0.448	0.390	0.339	0.440
C13	0.085	0.073	0.060	0.084	0.218	0.203	0.185	0.215	0.278	0.223	0.192	0.262	0.248	0.216	0.190	0.243
C14	0.111	0.091	0.068	0.109	0.182	0.155	0.120	0.180	0.216	0.164	0.137	0.205	0.236	0.202	0.162	0.233
C15	1.000	0.672	0.514	0.955	0.391	0.338	0.275	0.385	1.000	0.669	0.556	0.923	0.494	0.439	0.384	0.489

**Table 6:** Regression variables and results.

Dependent Variable	Description	Mean	SD	Coefficient by Dimension		
				Planning	Delivery	Quality
MRL	Medical Resource Level: a measurement of medical equipment required for radiation treatment delivery	7.68	4.20	0.2216 **	0.0055 *	0.4234 *
DIV	Radiation Treatment Diversification: Proportion of radiation treatments delivered to body regions other than pelvis or chest	30.50	10.12	9.2068 ***	−0.1373	−3.789
POP	Center Catchment Population: Population within 50 km radius of center (in ’000s)	585.93	350.10	−0.0007	0.0000	0.0000
TEA	Teaching hospital designation	See Table 1 for binary designation		−0.6216	0.0014	2.093
$R^{2}$				0.6364	0.0008	0.3846

*** *p* < 0.001, ** *p* < 0.01, * *p* < 0.05. Note: a positive (negative) coefficient indicates a decrease (increase) in efficiency.

## Data Availability

Ontario Health is prohibited from making the data used in this research publicly accessible if it includes potentially identifiable personal health information and/or personal information as defined in Ontario law, specifically the Personal Health Information Protection Act (PHIPA) and the Freedom of Information and Protection of Privacy Act (FIPPA). Upon request, data de-identified to a level suitable for public release may be provided by Ontario Health.

## Appendix A. Bootstrap DEA Model Solution Approach

### Appendix A.1. Input-Oriented VRS DEA Model

We follow the DEA frameworks established by [22,23], which are presented in [13]. We begin by detailing the primal linear program from which we construct the dual model used in the bootstrap DEA. Let c=1,2,3 denote the planning, delivery, and quality dimensions of the radiation treatment program, respectively. Let j=1,2,…,n denote the regional cancer centers (DMUs), $i=1,2,\ldots ,m^{c}$ be the inputs, and $r=1,2,\ldots ,s^{c}$ represent the outputs for each DMU. We use the following notation for every treatment dimension *c* and year *t*:

$\theta_{j}^{ct}=$ relative efficiency of DMU *j*

$x_{ij}^{ct}=$ input *i* from DMU *j*

$y_{rj}^{ct}=$ output *r* from DMU *j*

$v_{i}^{ct}=$ implicit price of input *i*

$u_{r}^{ct}=$ implicit price of output *r*

$u_{0}$ is an unrestricted in sign variable (urs)

For every DMU k=1,2,…,n; treatment dimension c=1,2,3; and year t=2013,…,2016, we have:

(A1) $$\begin{array}{ccc} {\left[P\right]}_{k}^{ct}\; & \max\; & \theta_{k}^{ct}=\sum_{r=1}^{s^{ct}}u_{r}^{ct}y_{rk}^{ct}+u_{0} \end{array}$$

(A2) $$\begin{array}{cccccccc} \; & \;s.t.\; & \sum_{r=1}^{s^{ct}}u_{r}^{ct}y_{rj}^{ct}-\sum_{i=1}^{m^{ct}}v_{i}^{ct}x_{ij}^{ct}+u_{0} & \leq 0 & \; & \forall j=1,2,\ldots ,n & \; & \end{array}$$

(A3) $$\begin{array}{cccccc} \; & \; & \sum_{i=1}^{m^{ct}}x_{ik}^{ct} & =1 & \; & \end{array}$$

(A4) $$\begin{array}{cccccc} \; & \; & u_{r}^{ct} & \geq 0 & \; & \forall r=1,2,\ldots ,s^{ct} \end{array}$$

(A5) $$\begin{array}{cccccc} \; & \; & v_{i}^{c} & \geq 0 & \; & \forall i=1,2,\ldots ,m^{ct} \end{array}$$

(A6) $$\begin{array}{cccc} \; & \; & u_{0} & \;urs \end{array}$$

Constraint (A2) ensures the sum of outputs for a DMU is less than or equal to the sum of its inputs, while (A3) scales the inputs of DMU to equal 1. Constraints (A4)–(A6) are defining domains of the variables. The objective function maximizes the total outputs of DMU *k*: if the result equals 1, then DMU *k* is considered efficient; otherwise it is deemed inefficient. (Note that we omit superscripts *c* and *t* from here on to streamline our notation, but all models are solved for every treatment dimension *c* and year *t*.)

An inefficient DMU need not despair: information regarding how it might reach the efficient frontier can be obtained from solving the dual of [P]. After introducing weights, $\lambda_{j}$ for DMU *j* and dual efficiency of DMU *k*, $\theta_{k}$, the dual of [P] is formulated as follows:

(A7) $$\begin{array}{ll} {\left[DEA\right]}_{k}\; & \min\;\theta_{k} \end{array}$$

(A8) $$\begin{array}{cccccccc} \; & \;s.t. & \sum_{j=1}^{n}x_{ij}\lambda_{j} & \leq x_{ik}\theta_{k} & \; & \forall i=1,2,\ldots ,m & \; & \end{array}$$

(A9) $$\begin{array}{cccccccc} \; & \; & \sum_{j=1}^{n}y_{rj}\lambda_{j} & \geq y_{rk} & \; & \forall r=1,2,\ldots ,s & \; & \end{array}$$

(A10) $$\begin{array}{cccccc} \; & \; & \lambda_{j} & \geq 0 & \; & \forall j=1,2,\ldots n \end{array}$$

(A11) $$\begin{array}{cccccc} \; & \; & \sum_{j=1}^{n}\lambda_{j} & =1 & \; & \end{array}$$

Here, we minimize the dual efficiency of DMU *k*, where Constraint (A8) ensures the weighted sum of reference inputs *i* is less than or equal to the input for DMU *k* and Constraint (A9) ensures the weighted sum of reference outputs *r* is greater than or equal to the output of DMU *k*. Inequalities (A10) ensure weights are non-negative.

An efficient DMU *t* will yield $\theta_{k}=1$. For an inefficient DMU *k*, solving [DEA] will give $\theta_{k}<1$. The reference set (or benchmarks) to this inefficient center is identified by observing the efficient DMUs $k^{\prime }=1,2,\ldots$ whose $\lambda_{k^{\prime }}>0$.

### Appendix A.2. Bootstrap DEA

We follow the approach outlined in [38], which uses the Shephard input δ (≥1) rather than the Farrell efficiency score θ (where θ≤1 and $\delta =\theta^{-1}$). For each year *t* and treatment dimension *c*:

(1) Solve an input-oriented VRS DEA and calculate $\delta_{j}$ for j=1,2,…,n.
(2) Let $\left\{\delta_{1},\delta_{2},\ldots ,\delta_{n},2-\delta_{1},2-\delta_{2},\ldots ,2-\delta_{n}\right\}$ be a list of mirrored efficiencies.
(3) For B=1,…,2000:
  (a) Generate a standardized realization of efficiencies through sampling with replacement from mirrored efficiencies;
  (b) Using this sample, generate fictional reference inputs, $x^{*}$;
  (c) Solve VRS DEA using original outputs and inputs, and use $x^{*}$ as reference inputs for DMUs 1,…,n.
(4) Calculate the bias-corrected mean efficiency, ${\hat{\theta }}_{j}$, and quantiles for each DMU *j*.

This bootstrap approach repeatedly samples inputs to generate a range of efficiencies, ultimately providing us with an *average* efficiency score that better distinguishes efficient DMUs centers from one another, even if that difference is small. Table 3, Table 4 and Table 5 show efficiency scores derived from [DEA], along with the bias-corrected mean efficiencies and their 5th and 95th quantiles (denoted by θ, $\hat{\theta }$, $q_{0.05}$, and $q_{0.95}$, respectively). The ranking of DMUs remains, in general, consistent with results from solving [DEA] just once.
